# Ambidexterity in Social Capital, Dynamic Capability, and SMEs’ Performance: Quadratic Effect of Dynamic Capability and Moderating Role of Market Orientation

**DOI:** 10.3389/fpsyg.2020.584969

**Published:** 2021-02-05

**Authors:** Luanping Zhou, Michael Yao-Ping Peng, Lijin Shao, Hsin-Yi Yen, Ku-Ho Lin, Muhammad Khalid Anser

**Affiliations:** ^1^Research Center of Open Economics and Trade, Fuzhou University of International Studies and Trade, Fuzhou, China; ^2^School of Economics & Management, Foshan University, Foshan, China; ^3^Department of International Business, Fuzhou Melbourne Polytechnic, Fuzhou, China; ^4^Department of International Business, Providence University, Taichung, Taiwan; ^5^Department of Business Administration, National Chung Hsing University, Taichung, Taiwan; ^6^School of Public Administration, Xi’an University of Architecture and Technology, Xi’an, China

**Keywords:** internal social capital, external social capital, ambidexterity, dynamic capability, market orientation

## Abstract

The importance of organizational ambidexterity was stressed in different fields of management. This study was using a distinct method to measure the differences in the degree of ambidexterity to bridge the gap with the previous studies and to provide more insights in the successful management of exploitation and exploration. This study surveyed Taiwanese small and medium-sized enterprises (SMEs) to test the hypotheses. We issued 1000 questionnaires in total and received 234 valid ones. Results indicate exploitative and explorative capabilities exerting non-linear effect on performance. Likewise, ambidexterity and its interaction with market orientation have positive influence on firm performance. This study used structural equation modeling to analyze data, as this approach is known to be particularly advantageous for the exploratory nature of this study. We also used hierarchical regression analysis to test interaction and moderating effects. The study contributes to the literature in two ways. First, we offer a clearer understanding of the complete concept of social capital, including inter-firm and intra-firm social capital, and how contributes to improving and extending existing exploitative capabilities. Second, this study outlines how market orientation can have different effects on an ambidextrous strategy that is adopted to improve Taiwanese SMEs performance.

## Introduction

Over recent few decades, small and medium-sized enterprises (SMEs) have been among the most momentous in the development of economy. With the intense increase in competitive forces in both domestic and global markets, and rapid changes in the technology and environment, firms now need to continually accumulate, update and renew their own knowledge and core competence (Costello and McNaughton, 2018). In such cases, dynamic capabilities (DCs) become important, because, as Morgan (2012, p. 108 note, “the firm’s ability to engage in market-based learning and use the resulting insight to reconfigure the firm’s resources and enhance its capabilities in ways that reflect the firm’s dynamic market environment.” However, problems concerning resource allocation will occur when dynamic capabilities are divided into two or more capability orientations (Drnevich and Kriauciunas, 2011). Valuable exploration and exploitation may become liabilities when firms face environmental turbulence (O’Reilly and Tushman, 2008; Costello and McNaughton, 2018), especially if capability gaps (trade-off relationship) arise (Day, 2011; Drnevich and Kriauciunas, 2011). Firms that only focus on exploration or exploitation may then face unforeseen threats to their competitive advantage (Rialti et al., 2019), such as core rigidity, competence traps, and so on (Andriopoulos and Lewis, 2009; Drnevich and Kriauciunas, 2011). As such, this study aims to apply the concept of ambidexterity from the complementary view of exploration and exploitation to explore correlations between DCs and firm performance in Asian SMEs (He and Wong, 2004; Menguc and Auh, 2008; Cao et al., 2009; Costello and McNaughton, 2018; Rialti et al., 2018). With regard to firm performance, Rialti et al. (2018) illustrated that ambidextrous organizations devote themselves to pursuing dynamic business process management and strengthening organizational flexibility, enabling the firms to identify eventual inefficiencies in internal processes and adjust production quantity addressing to the customer need (Rialti et al., 2019). This study also adopts organizational effectiveness, growth/share and profitability to measure firm performance.

In addition to illustrating methods that can be used to develop capabilities (Subramaniam and Youndt, 2005) and explore the influence of capabilities on firm performance and survivability, several studies note that the “resource-capabilities-competitive advantage (performance)” relationship is a continuous development process, as a resource produces capabilities and capabilities further influence firm performance (Dyer and Hatch, 2006; Andersson et al., 2007). Although most literatures that look at enhancing DCs were intensively focused on the application of internal resources or the knowledge integration process, few of them discussed such issues from the perspective of an intangible relationship factor (i.e., social capital) (Adler and Kwon, 2002; Andersson et al., 2007). Most scholars engaged in the studies of management theory have been attracted by the organizational learning theory during the process of knowledge integration, resulting in the theory which is enriched in the combination of different knowledge domains (Liao et al., 2016). According to Blyler and Coff (2003), with the help of facilitating a continuous flow of information from various sources that are both external and internal, social relationships among manufactures do promote the development of new capacities (McEvily and Zaheer, 1999; Dyer and Hatch, 2006; Pinho and Prange, 2016). Rialti et al. (2018) have proposed organizational agility and indicated that market capitalization and operational adjustment agility are two fundamental outcomes of the pursuit of agility which were be strengthened via internal and external evidences. Most firms turn to new knowledge acquisition from outside or new knowledge learning from partner enterprises, so as to avoid the imitation and replication of competitors (Rakthin et al., 2016), as well as achieving the efficiency promotion of internal process or new products development (Liao et al., 2016). Firms should establish strategic relationships with key partners and work to leverage the valuable knowledge assets obtained from their strategic relationships to enhance DCs (Nahapiet and Ghoshal, 1998; Adler and Kwon, 2002; Yli-Renko et al., 2002; Griffith and Harvey, 2004; Dyer and Hatch, 2006; Francis et al., 2009). Despite these insights from earlier works, how Taiwanese SMEs’ social capital drives the development of DCs (exploration and exploitation), and how social capital and DCs affect firm performance, remain under-researched (Pinho and Prange, 2016). Therefore, this study aims to explore social capital as a significant antecedent for the development of DCs.

While most studies have verified that organizational ambidexterity has positively related to performance (He and Wong, 2004; Cao et al., 2009; Raisch et al., 2009), some scholars argue that it has a negative influence (Menguc and Auh, 2008). For example, Kristal et al. (2010) claimed that due to the limitations of resources and managerial scope, there is a trade-off between exploration and exploitation instead of a complementary relationship when an ambidextrous approach of adopted. However, few studies have determined whether there are varying effects on exploration and exploitation in different cultural contexts (Lin et al., 2016; Peng et al., 2019a, b). Kristal et al. (2010) stressed the trade-off relationship between the two capabilities, while Tushman and O’Reilly (1997) reported that an ambidextrous approach may succeed when there is a strong form of social control and common culture connecting the two capabilities (Gibson and Birkenshaw, 2004; Andriopoulos and Lewis, 2009). Menguc and Auh (2008) proposed that market orientation is referred as a complete organizational culture that can integrate exploration and exploitation within the firm, and thus make them have a complementary, instead of a trade-off, relationship. Based on above arguments, this study aims to explore moderating role of marketing orientation between ambidexterity and firm performance.

Overall, this study makes four main contributions to existed literatures. Firstly, unlike previous works that have emphasized the maintenance or establishment of external social capital, we offer a clearer understanding of the complete concept of social capital, including inter- and intra-firm social capital, and this is the main aim of this work. Second, this study investigates how social capital contributes to improving and extending existing exploitation, and to renewing and modifying exploration, with the goal of enhancing Taiwanese SMEs performance. Third, we provide specific empirical insights regarding the impact of ambidexterity and its complementary role in aligning exploration and exploitation. Finally, this study outlines how market orientation can have different effects on an ambidextrous strategy that is adopted to improve Taiwanese SMEs performance, and investigates whether a market orientation mechanism can effectively integrate exploration and exploitation to make such a strategy more successful.

According to our research purposes, this study firstly introduces relevant literature about social capital, dynamic capability, ambidexterity and market orientation and develops several hypotheses. Moreover, we conduct a survey to investigate Taiwanese SMEs’ information and provide well-established scales. Besides, this study adopts PLS-SEM to verify measurement model and structural model via using Smart PLS 3.0, and then discuss research finding and provide implications in the Conclusion section.

## Literature Review and Hypotheses Development

### Social Capital: Inter- and Intra-Firm

Research on inter-firm relationships and networks is focused on deep interactions (based on resources, friendship and information) across multiple networks (such as individuals, teams, and organizations) (Lin et al., 2016; Mazzola et al., 2016). Scholars have not yet reached an agreement on measuring the variables of inter-firm social capital (McEvily and Marcus, 2005; Tsai et al., 2009).

Nahapiet and Ghoshal (1998) illustrated that the connection of members’ use relies on ideas sharing, which may make new knowledge come into being rather than doing current information transfer (Zaheer and Bell, 2005; Lin et al., 2016). Firms could thus access and acquire more new knowledge and creative ideas from interactions with their members, which could then be applied to foster internal and external innovation (Mazzola et al., 2016), so as to improve their exploitation and exploration (Dyer and Hatch, 2006).

Some scholars claimed that joint problem solving within network facilitates the development of competitive capabilities, with parties sharing the responsibility to maintain their relationship in order to overcome common problems (McEvily and Marcus, 2005; Andersson et al., 2007). This kind of cooperation agreement contains joint mechanisms that are following by both parties. Whenever a problem arises, firms will seek joint problems solving through coordination (Lin et al., 2016; Mazzola et al., 2016). Firms engaging in joint problem solving are also likely to establish a high degree of trust. Partners may thus be willing to make additional efforts to help solve problems with more exploitation and exploration learning processes (Atuahene-Gima and Murray, 2007), which not only can reduce the risk of information asymmetry in the transaction process, but also help firms to share tacit and explicit knowledge and increase the transparency of information exchanges (Dhanaraj et al., 2004).

Tiwana (2008) declared that, on the basis of shared values from the members on the Internet, manufactures can draw ideas from each other (Reagans and McEvily, 2003) that conduces to tacit knowledge transfer and integration, makes distrust and uncertainty deduction, as well as mutual coordination acceleration, and facilitates problem solving (McEvily and Marcus, 2005; Andersson et al., 2007; Lin et al., 2016). If a high degree of intra-firm cognition and social identity exists, strong ties will be created to enhance reciprocal knowledge acquisition and reduce the demand for formal supervision, and thus SMEs could focus more efforts on knowledge absorption and application (Tsai and Ghoshal, 1998; Presutti et al., 2007). For this brief review of the literature, it can be seen that joint problem solving, bridging ties and shared values can all help middle and small-sized enterprises to accumulate the knowledge sources required to improve their abilities of both exploration and application. Through inter-firm interaction and communication, external knowledge, information and resources can be transformed into the basis of internal capability cultivation, as stated in the following hypotheses:

*H1a:* Inter-firm social capital positively correlates with exploration.
*H1b:* Inter-firm social capital positively correlates with exploitation.

Intra-firm social capital can be conceptualized by considering all of the members in a firm as nodes and the interactions between them as ties (Paruchuri and Awate, 2017), and an appropriate intra-firm structure will facilitate firms to acquire useful information and knowledge from interactions (Nahapiet and Ghoshal, 1998; Lin et al., 2016), so that they can quickly establish new technical knowledge (Tsai and Ghoshal, 1998; Andersson et al., 2007; Paruchuri and Awate, 2017) and improve their capabilities of combining existing knowledge (Andersson et al., 2007; Li, 2004). According to the model presented in Nahapiet and Ghoshal (1998), the current study proposes that intra-firm social capital consists of structural, cognitive and relational factors, and discusses key constituents of internal social capital, such as information-based mechanisms (structural), people-based mechanisms (structural), trust (relational), and shared vision (cognitive), based on the work of Tsai and Ghoshal (1998), Yli-Renko et al. (2002), and Tsai et al. (2009).

Information-based mechanisms can effectively deal with majority of information and provide information in a timely manner to facilitate communication among firms, thus to ensuring the information transferring, flowing, and sharing (Hartmann et al., 2008). People-based mechanisms can be used to obtain external knowledge, and the organization depends on internal members to transform any new knowledge into management practice (Tsai et al., 2009). The existence of effective people-based mechanisms can help the interpersonal communication that is needed among members (Kim et al., 2003). In other words, well-established communication facilitates to integrate the existing knowledge and information in shaping several outcomes of consequence, and then interpret this knowledge, endow it a certain meaning, and transform it into organizational routines (Yli-Renko et al., 2002; Li, 2004; Tsai et al., 2009; Paruchuri and Awate, 2017).

Trust is omnipresent in the economic system, no matter for personal relationships or economic development. Trust is also an important factor that determines the relationship quality. Trust is a manifestation of willingness to believe in and rely on partners (Morgan and Hunt, 1994). Psychologists define trust as an expectation that the commitment of the other party is reliable (Blomqvist et al., 2005). Trust can also be defined as the willingness to take the risk of being harmed by the expected specific behavior of the other party (Tsai and Ghoshal, 1998). Firms believe that both parties in the partnership will take actions that are beneficial to each other, instead of unexpected negative actions; they will take into account the maximum benefit of the alliance, and their behaviors are consistent and not opportunistic (Young-Ybarra and Wiersema, 1999). Previous studies indicated that high levels of trust among partners can facilitate the achievement of the goal of information sharing (Gupta and Govindarajan, 2000; La Londe, 2002). Trust relationship may be based on previous experience and interactions with specific partners. Previous informal relationships can reduce the search costs of partners and enhance the sense of trust (Ahuja, 2000). Besides, a base of common knowledge is to be established if employees can do efficient communication and share knowledge with each other, and such a base of common knowledge can promote the incorporation of diversified knowledge in order to make efficient application and new ideas creation. When members share common vision, they will more willing to understand each other’s behaviors, and thus misunderstandings will be reduced and ideas and recourses can be exchanged more easily (Tsai and Ghoshal, 1998). From this review of earlier studies it is clear that middle and small-sized enterprises should work to establish and maintain strong internal social capital, as this can help members to deliver, transfer and apply external information and knowledge, and thus to adjust the firms’ dynamic capabilities and respond to environmental changes more flexibly. This is stated in the following hypotheses:

*H2a:* Intra-firm social capital positively correlates with exploration.
*H2b:* Intra-firm social capital positively correlates with exploitation.

### Relationship Among Exploration, Exploitation, and Firm Performance

The emphasis of exploration lies in new alternatives inspection (March, 1991; Peng et al., 2019b), which can be an origin that new technology, knowledge (Rothaermel and Alexandre, 2009; Lin et al., 2016) and innovation capacity (Peng et al., 2019a) are derived from. Then the exploration can be regarded as the basis of organizational growth. Manufactures which are moving in competitive surroundings and lacks resources and industrial development will be dedicated to searching chances for acceleration and innovation growth (Peng et al., 2019a). As for enterprises’ internationalization, Prange and Verdier (2011) stated that exploration in manufactures is considered as a flexible way of taking advantage of value-added or disruptive capacities so as to gain new and innovative competitive edge. Overall, a disruptive capability can increase the tendency for organizations to engage in actions that changes the basic structures they operate within, letting them overcome problems of path-dependence and organizational inertia and thus expedite firm growth (Peng et al., 2019a, b). As such, exploration cannot merely create new products as well as develop new markets (Jansen et al., 2006), but also enable firms to design more suitable organizational structure (He and Wong, 2004).

However, only focusing on exploration can lead to problems, as this will then consume resources that could also be used for the exploitive capabilities, and this uneven approach can cause substantial experimental costs and even losses (March, 1991; Peng et al., 2019a, b). With their capital invested in development of new knowledge and/or technology, firms that over-engage in their exploration may fail to achieve sufficient net profits to maintain business operation. The establishment of the exploration and implementation of related activities often requires more time than needed for the exploitation activities. Moreover, developing the exploration requires certain risks and costs due to the related uncertainties (Andriopoulos and Lewis, 2009). When an enterprise assigns excessive resources to exploration, its performance may be negatively affected, rather than positively. Exploration-oriented firms may launch products that are not technically accessible and not acceptable for customers, or assign too many resources to ideas that are not yet mature (Stock and Reiferscheid, 2014). Saad and Zantout (2014) also found that some large firms have overinvested in R&D, resulting in an adverse effect on the enterprise performance. According to the principle of diminishing resource returns in economics, the use of resources bring the benefits of increasing output at the beginning, but when the amount of resources reaches a certain level, the output may decrease if the resources are further added. This is because other production conditions are not changed in the short term, and there are not other matching resources. In other words, the performance will be decreased due to the increase of resource input (Slotegraaf et al., 2003), then this may reduce their chances of survival (Prange and Verdier, 2011). Therefore, we hypothesize:

*H3:* Exploration has a non-linear relationship with firm performance.

Exploitation puts emphasis on the short-term profits. It refers to the extension or added value of existing knowledge, information and technology, and signifies the characteristics of improvement, efficiency, productivity, execution and selection. Firms mainly develop existing markets, and thus exploitative actions, until they accumulate adequate capabilities. By reducing the uncertainty related to explorations and experiments, firm survivability can thus be improved in this manner (Prange and Verdier, 2011). Slater and Narver (1995) proposed that firms which engage in continuous learning will tend to track and respond to consumer demands in a more effective manner, identifying and capturing market opportunities and thus promoting profitability, sales growth, and customer retention (Lin et al., 2016). Manufactures can be conscious of how to avoid repeated mistakes, keep down production and transaction expenses, and enhance the capacity of dealing with a problem by accumulating learning experience (Jiang and Li, 2009). Nevertheless, an organization might suffer from growth deduction or be out of date on account of technological advancement or vicissitudes in customer preferences if it is immersed in exploitation (Levinthal and March, 1993). And along with time, the current routines in an organization are extracted and conducted, and with powerful subjective learning, an organization frequently adopts the old routines that are the same (Peng et al., 2019a), so that it cannot meet a new situation or environment, is likely to follow the wrong development path (Levinthal and March, 1993; Stock and Reiferscheid, 2014). According to claims of Prange and Verdier (2011), firms with a focus on exploitation may achieve organizational survival in the short-term by effectively utilizing existing resources and knowledge, but in the long term they are likely to fail to grow and thus decline. Therefore, this study proposed following hypothesis:

*H4:* Exploitation has a non-linear relationship with firm performance.

### Effect of Organizational Ambidexterity on Firm Performance

As for the concept of ambidexterity, which Gibson and Birkenshaw (2004) referred to the findings of Tushman and O’Reilly (1996), it intends to be companies that can do both rapid and flexible exploration conduction in emerging markets relying on new products or services development (Andriopoulos and Lewis, 2009). The concept of ambidexterity represents the combination of exploration and exploitation, with these not having a competitive, trade-off relationship, but stead one that allows them to complement each other (He and Wong, 2004; Gupta et al., 2006; Cao et al., 2009). Relying on exploration and exploitation, managers can detect relevant knowledge/resources in an easier way by means of current knowledge and resources and comprehend the situation more thoroughly, then they can achieve more effective reconfiguration of current knowledge/resources when they go through the development of new products and markets by means of promoting exploration and exploitation (Cao et al., 2009). The ambidextrous term formed by exploration and exploitation will help SMEs focus on the development of knowledge acquisition and knowledge creation simultaneously, and strengthen allocation of knowledge and resource (Heavey et al., 2015; Broersma et al., 2016; Vahlne and Jonsson, 2017; Luca et al., 2018). Furthermore, several scholars claimed that the complementary perspective can be referred as an organizational structure (Tushman and O’Reilly, 1996; Andriopoulos and Lewis, 2010; Peng and Lin, 2019; Peng et al., 2019a), which promotes firms to establish various organizational structures to engage in activities with exploration and exploitation in the organizational learning process. Rialti et al. (2018) claim that “ambidexterity, in fact, has been deemed related to both increase in organization agility and performance” (p. 1092). Manufactures can gain competitiveness reinforcement when they do external knowledge internalization by means of exploration, putting the processes of efficient administration into use in economy of larger scale (Peng et al., 2019a). The synergy between new opportunities and the limit on current routines and knowledge can be leveraged by organizational ambidexterity (Rialti et al., 2019), which is stressed by us. According to Miner et al. (2001), during the improvisation, current elements which are restructured with new methods conduce to connection of proper ideas to certain needs in the meantime (Peng et al., 2019a). Thus, this study proposed following hypothesis:

*H5*: The strength of organizational ambidexterity positively correlates with firm performance.

### Moderating Effect of Market Orientation

According to Narver and Slater (1990), “market-oriented” is a composition culture which do efficient and effective value creation for customers, leading to superior company performance establishment. There are three aspects of cultural measurement proposed: (1) “customer orientation,” meaning that, on the basis of employment, students can figure out the requirements and expectations from future employers; (2) “competitor orientation,” which analyzes the perception of the short-term advantages and disadvantages, as well as long-term potential capabilities and strategies of development, from the current and potential graduates in other universities; (3) “inter-functional coordination,” which illustrates that, by virtue of integrating and applying on-campus curriculum and administrative resources (Anwar, 2008), the university can create the value of future superior employees (i.e., graduates), which is contributed to the target employer (Anwar, 2008). Based on powerful social control and a combination of common culture and capacities of an organization, ambidexterity is more likely to succeed (Gibson and Birkenshaw, 2004; Peng et al., 2019a). Menguc and Auh (2008) demonstrated that the market orientation is regarded as the organizational culture with exploration and exploitation united as one, which are complementary instead of trade-off. Most empirical studies, not only from the perspective of culture, but also from an integrated view, have had a discussion on the meaning of organizational culture, as well as the strategic behavior (Homburg and Pflesser, 2000; Peng et al., 2019a), which illustrate that effective behavior can be accelerated by market orientation on the basis of accumulating, sharing and replying to correlative information from customers and contenders. Moreover, as manufactures carry out a kind of culture that can be shared among different departments and do adoption of valuable resources in a manner of harmony, they can enhance production efficiency via the enhancement of interdepartmental communication, cooperation and coordination (Narver and Slater, 1990; Peng et al., 2019a). Market orientation strikes a complementary between exploration and exploitation while fostering a shared organizational atmosphere, enabling both capabilities to work toward creating and delivering superior customer value (Menguc and Auh, 2008). We thus hypothesize the following:

*H6*: Market orientation positively moderates the relationship between organizational ambidexterity and performance.

**FIGURE 1:**
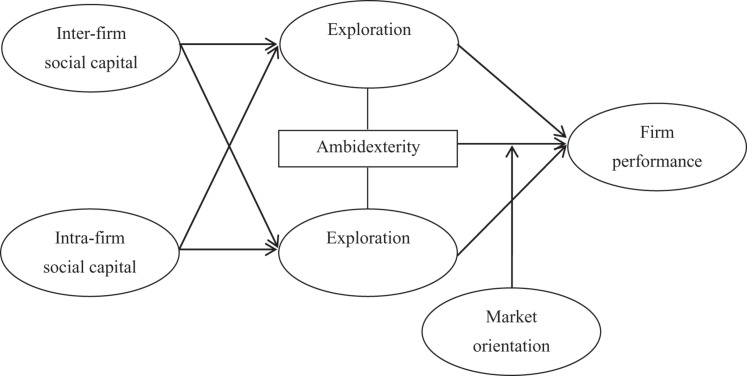
Conceptual framework.

Building on the above arguments, the authors thus present Figure 1.

## Methodology

### Sample and Procedure

We employed Taiwanese SMEs to test the hypotheses, and surveyed informants’ (CEOs per 16%, vice presidents per 27%, senior managers per 57%) knowledge about their companies and their relationship with stakeholders. As these managers should be familiar with the actual status of their firms’ partnership, development of capability and operations, this study selected them as the main information providers. A questionnaire was issued in October and November 2019. We sent 1,000 questionnaires and received 237 completed surveys, giving a 23.7% response rate. There were 234 valid questionnaires after eliminating three invalid responses, giving a 23.4% effective response rate. Table 1 summarizes the respondents’ demographic characteristics.

**TABLE 1:** Demographic characteristics of respondents.

Characteristics		Frequency	Ratio
Industrial sector	Motor manufacturing	83	35.47
	Electronic parts	45	19.23
	Chemicals	23	9.83
	Semiconductors	17	7.26
	Precision machinery	17	7.26
	Information technology	39	16.67
	Other	10	4.27
Profitability	Low profit	113	48.29
	Medium profit	60	25.64
	High profit	62	26.50
Firm age	Lower than 5 years	219	93.59
	5–10 years	68	29.06
	10–15 years	58	24.79
	15–20 years	55	23.50
	More than 20 years	34	14.53
Firm size	Lower than 50 employees	23	9.83
	50–100 employees	62	26.50
	100–150 employees	58	24.79
	150–200 employees	46	19.66
	More than 200 employees	45	19.23

This study hid the names of constructs, and assigned the question items randomly to prevent common method variance (CMV). The Harman one-factor analysis method as used to test for CMV. The explained variance in one factor was 43.52%, which is smaller than the recommended threshold of 50%. Therefore, CMV was not problematic in this study (Podsakoff and Organ, 1986; Podsakoff et al., 2003, 2012).

### Measurement

The variables examined in this study were chiefly developed from scales presented in the existing literature. Except for firm size and firm age, all items were using a seven-point Likert scale. The five items on the exploration and the four items on the exploitation were taken from He and Wong (2004); Lubatkin et al. (2006), Cao et al. (2009), and Lin et al. (2016), and Peng et al. (2019a; 2019b).

Following Gibson and Birkenshaw (2004) and Peng et al. (2019a; 2019b), the concept of ambidexterity used in this study, a multiplicative term of exploration and exploitation. Since we measured ambidexterity as the multiplication of exploration and exploitation, we acknowledged that it may suffer from multicollinearity. To minimize this concern to our analyses, we mean-centered exploration and exploitation before deriving ambidexterity. The score of the ambidexterity is mainly the value of multiplicative term of exploration and exploitation.

Inter-firm social capital refers to the research results of Dhanaraj et al. (2004) and McEvily and Marcus (2005), this study takes bridging ties (three items), joint problem solving (three items), and shared values (four items) as variables to measure inter-firm social capital. Intra-firm social capital was adopted from Tsai and Ghoshal (1998), Yli-Renko et al. (2002), and Tsai et al. (2009) which was measured in terms of information-based mechanisms (five items), people-based mechanisms (five items), trust (five items), and shared vision (two items).

Firm performance is a complex construct. Following Narver and Slater (1990), Slater and Narver (1994), Jaworski and Kohli (1993), and Lubatkin et al. (2006), this study adopted multi-dimensional methods to measure firm performance: organizational effectiveness (three items), growth/share (three items), and profitability (three items).

Consistent with previous work in the marketing literature (Kohli and Jaworski, 1990; Narver and Slater, 1990; Anwar, 2008; Menguc and Auh, 2008), we operationalized market orientation as a higher-order construct of customer orientation (seven items), competitor orientation (five items), environmental scanning (five items), strategy implementation (four items), and new services development (four items).

This study controlled firm size and firm age that might affect the model. Since firm age expresses a firm’s development stage, and is associated with its exploration and exploitation (Cao et al., 2009), these attributes also served as control variables. All scales were shown in Appendix 1.

#### Data Analysis Strategy

This study tested the hypotheses of the research framework and included paths via structural equation modeling. The hypotheses of the research framework are tested and paths are included in this study via structural equation modeling. For higher-order constructs (internal social capital, external social capital, dynamic capability, firm performance, and market orientation), we reduced the number of parameters which are to be estimated following the partial aggregation method. This procedure involves averaging the responses of subsets of items measuring a construct. As internal social capital, external social capital, dynamic capability, firm performance, and market orientation was multi-dimensional constructs, we averaged responses of each dimensions to serve as indicators for these constructs. Construct validity analysis was performed using IBM-AMOS statistical program, v. 23.0 for Windows. Partial least squares structural equation modeling (PLS-SEM) was adopted to construct the structural model; specifically, verification of the structural model was performed using SmartPLS 3.0 (path analysis).

## Results

### Reliability and Validity

This study adopts confirmatory factor analysis (CFA) using AMOS 23.0 to measure and also takes into consideration of the criteria of convergent validity set by Hair et al. (2010), that is, (1) All the standardized factor loadings must be greater than 0.5 and reach the level of significance (2) the value of composite reliability (CR) must be higher than 0.7 (3) the average variance extracted (AVE) must exceed 0.5. As all the coefficients of the factor loadings of measured variables in this study are great than 0.5, and all the measured variables are significant, so the measurement model studied has considerable convergent validity. What is more, the CR and AVE values of the variables in this study range from 0.78∼0.91 and 0.50∼0.68 respectively, and all the variables showed a good fitness, indicating the good convergent validity between the variables in this measurement mode (shown in Table 2). All three criteria for convergent validity were met, and correlation coefficients were all less than the square root of the AVE within one dimension, suggesting that each dimension in this study had good discriminant validity.

**TABLE 2:** Descriptive statistics and correlations.

	1	2	3	4	5	6	7	8	9	10	11	12	13	14	15	16	17
(1) Bridging ties	**0.74**																
(2) Joint problem solving	0.54	**0.63**															
(3) Shared value	0.44	0.39	**0.74**														
(4) Information-based mechanism	0.54	0.38	0.51	**0.81**													
(5) People-based mechanism	0.39	0.19	0.27	0.70	**0.7**												
(6) Trust	0.40	0.30	0.31	0.58	0.56	**0.72**											
(7) Shared vision	0.18	0.18	0.22	0.30	0.22	0.46	**0.67**										
(8) Exploration	0.53	0.21	0.30	0.53	0.56	0.58	0.28	**0.79**									
(9) Exploitation	0.26	0.09	0.07	0.28	0.31	0.19	0.10	0.55	**0.69**								
(10) Organizational effectiveness	0.25	0.35	0.02	0.30	0.21	0.24	0.42	0.26	0.31	**0.81**							
(11) Growth/share	0.28	0.26	0.07	0.25	0.21	0.19	0.30	0.29	0.32	0.78	**0.8**						
(12) Profitability	0.37	0.22	0.32	0.41	0.35	0.25	0.24	0.36	0.27	0.53	0.66	**0.8**					
(13) Customer orientation	0.31	0.30	0.28	0.36	0.30	0.43	0.61	0.41	0.34	0.53	0.48	0.46	**0.83**				
(14) Competitor orientation	0.28	0.22	0.28	0.28	0.23	0.53	0.62	0.41	0.18	0.43	0.37	0.37	0.83	**0.79**			
(15) Environmental scanning	0.29	0.27	0.24	0.34	0.26	0.48	0.61	0.39	0.23	0.55	0.45	0.40	0.78	0.87	**0.74**		
(16) Strategy implementation	0.35	0.31	0.30	0.40	0.28	0.44	0.45	0.45	0.20	0.44	0.43	0.41	0.63	0.67	0.72	**0.71**	
(17) New services development	0.28	0.34	0.05	0.38	0.28	0.42	0.39	0.36	0.30	0.83	0.65	0.46	0.44	0.42	0.50	0.48	**0.71**
Cronbach’α	0.77	0.73	0.83	0.90	0.87	0.81	0.76	0.89	0.77	0.87	0.80	0.87	0.90	0.89	0.81	0.82	0.79
CR	0.78	0.83	0.83	0.89	0.82	0.81	0.62	0.89	0.83	0.85	0.80	0.88	0.91	0.89	0.83	0.79	0.80
AVE	0.55	0.52	0.55	0.66	0.50	0.52	0.45	0.62	0.51	0.59	0.58	0.66	0.68	0.62	0.55	0.50	0.50

*Square root of AVE for each latent construct is given in diagonals.*

### Testing Structural Model Fit

Before proceeding to examine the structural model, we first tested model fit. Henseler et al. (2015) proposed three model fitting parameters: the standardized root mean square residual (SRMR), the normed fit index (NFI) and the exact model fit. According to Henseler et al. (2015), the evaluation standards for convergent validity are (1) NFI should be larger than 0.9, (2) SRMR should be less than 0.08, (3) exact model fit, which tests the statistical (bootstrap-based) inference of the discrepancy between the empirical covariance matrix and the covariance matrix implied by the composite factor model. Dijkstra and Henseler (2015) suggested the *d_LS* (squared Euclidean distance) and *d_G* (geodesic distance) as two different ways to compute this discrepancy. Henseler et al. (2015) indicated that *d*_*ULS*_ and *d*_*G*_ < than the 95% bootstrapped quantile (HI 95% of *d*_*ULS*_ and HI 95% of *d*_*G*_).

In this study, the SRMR value was 0.056 (<0.08) and the NFI was 0.941 (>0.90) and the *d*_*ULS*_ < bootstrapped HI 95% of *d*_*ULS*_ and *d*_*G*_ < bootstrapped HI 95% of *d*_*G*_ indicating the data fits the model well.

### Inner Model Analysis

This study used partial least squares structural equation modeling (PLS-SEM) to analyze data to test the direct effects of the model, as well as test the model’s explanatory power (*R*^2^) regarding the frequency of inter- and intra-firm social capital processes having effects on exploration and exploitation. To assess the structural model, Hair et al. (2017) suggested looking at the *R*^2^, beta (β) and the corresponding *t*-values via a bootstrapping procedure with a resample of 5,000. Prior to hypotheses testing, the values of the variance inflation factor (VIF) were determined. The VIF values were less than 5, ranging from 1.392 to 2.299. Thus, there were no multicollinearity problems among the predictor latent variables (Hair et al., 2017).

**FIGURE 2:**
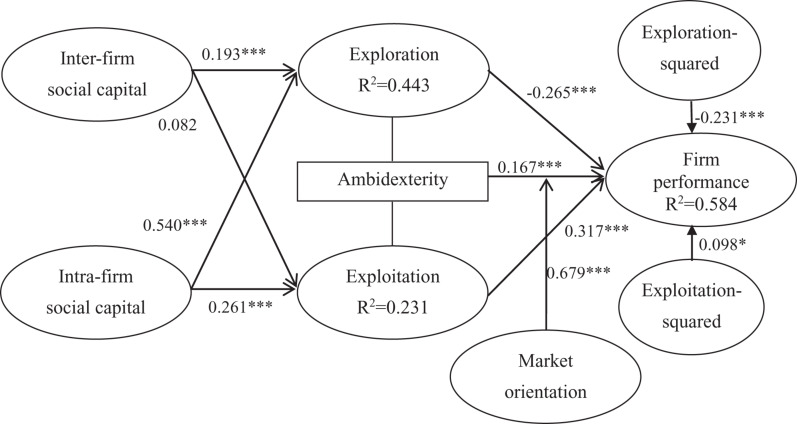
Main path analysis. ^∗^*p* < 0.1; ^∗∗∗^*p* < 0.01.

Next, we examined the path coefficients and their significance values to test the hypotheses, and used PLS-SEM to analyze data (Tsang, 2002). The results of H1a and H1b suggest that inter-firm social capital has a significant, positive relationship with exploration (β = 0.193, *p* < 0.01), but does not relate to exploitation (β = 0.082, *p* > 0.10). Therefore, the findings support H1a, but reject H1b. The results of H2a and H2b indicate that intra-firm social capital relates significantly and positively to exploration (β = 0.540, *p* < 0.01) and exploitation (β = 0.261, *p* < 0.01). Therefore, the findings support H2a and H2b. Moreover, PLS-SEM results, presented in Figure 2 and Table 3, show that the quadratic term of exploration is negatively and significantly related to firm performance (β = −0.231, *p* < 0.01), which supports H3 and shows an inverted U-shaped relationship. The quadratic term of exploitation is positively and significantly (β = 0.098, *p* < 0.1) related to firm performance, which supports H4 and shows a U-shaped relationship.

**TABLE 3:** Results of the paths.

Hypotheses	Std. β	*t*-value	Significance CI (2.50-97.5%)	VIF	*f*^2^
**Direct Paths**					
H1a: Inter-firm social capital → Exploration	0.193***	2.673	(0.047∼0.342)	1.424	0.047
H1b: Inter-firm social capital → Exploitation	0.082	0.834	(−0.108∼0.265)	1.424	0.005
H2a: Intra-firm social capital → Exploration	0.540***	7.736	(0.406∼0.682)	1.424	0.367
H2b: Intra-firm social capital → Exploitation	0.261***	2.730	(0.406∼0.682)	1.424	0.053
H3: Exploration^2^ → Firm performance	−0.231***	4.952	(−0.319∼−0.137)	2.299	0.123
H4: Exploitation^2^ → Firm performance	0.098*	1.884	(−0.036∼0.169)	1.707	0.036
H5: Ambidexterity → Firm performance	0.167***	2.903	(0.063∼0.302)	2.105	0.042
**Moderating Effect**					
H6: Ambidexterity × Market orientation → Firm performance	0.679***	13.679	(0.583∼0.779)	1.392	0.111

*p < 0.1; ***p < 0.01.

Finally, Figure 2 and Table 3 indicates that ambidexterity is significantly and positively related to firm performance (β = 0.167, *p* < 0.01). Consequently, H5 was supported. With regard to H6, which posits that the positive effect of ambidexterity on firm performance gets reduced by greater market orientation, Table 2 shows that the interaction term between market orientation and ambidexterity has a positive moderating effect on firm performance (β = 0.679, *p* < 0.01). The relationship between ambidexterity and firm performance becomes significantly more positive when the level of market orientation is high. Therefore, our finding supports H6.

## Conclusion

### Discussion

On the basis of organizational learning and the opinion of dynamic capacity, the study is implemented for an establishment of a complete research model, and according to which, the organizational ambidexterity is found to be a kind of dynamic capacity, and it is featured with path dependence. Through developing our research model, we further understand nature of dynamic capability and discuss the antecedents and consequences of dynamic capability on the basis of organizational learning theory, bringing more insights and discussions to the development of dynamic capability. Cultivating and developing the exploration and exploitation of ambidexterity depends on the stock of resources and knowledge that an enterprise has (Eisenhardt and Martin, 2000). From the social capital view, this study discussed how a firm should establish and maintain relationships with its partners and how it should work to formal and informal mechanisms with internal members to achieveambidexterity with regard to exploration and exploitation. Moreover, when there is strong social control and a common culture to connect these two capabilities, ambidexterity will have an even greater influence. Therefore, this study examined the contextual effect of market orientation on ambidexterity.

This study discusses the relationships among social capital, dynamic capability, ambidexterity, market orientation, and firm performance, which has extended recent dynamic capability and organizational learning research (He and Wong, 2004; Menguc and Auh, 2008; Cao et al., 2009; Kristal et al., 2010; Costello and McNaughton, 2018). Specifically speaking, this study makes the following contributions. First, the results showed that inter-firm social capital and intra-firm social capital are conducive to enhancing the quality and quantity of knowledge acquisition and exchange through external and internal cooperation, so as to help a firm develop both new and existing exploration and exploitation. As stated in previous studies (e.g., Peng et al., 2019a, b), is verified to be true through the statistical analysis in the present study in an Asian context. This is a supplement to the previous opinions and consolidates the support for the social capital dynamic capability via organizational learning perspective (Aranda et al., 2017). Moreover, the results of the current study showed that inter-firm social capital did not have any influence on exploitation. This is inconsistent with McEvily and Marcus (2005); Andersson et al. (2007), and Lin et al. (2016), who found significant effect of social capital on capability. However, this is consistent with claims proposed from Wang et al. (2015), in particular, noting that dynamic capability associated with knowledge process contains a wider range of knowledge, resource, routines et, also including exploitation. In other words, SMEs who invest heavily in a single partner in order to obtain knowledge may confront problems of high repeatability and low value, and may fail to use existing knowledge stores, which then become a sunk cost.

Most scholars believe that exploration and exploitation have a significant influence on firm performance, such as improving long-term performance (Tushman and O’Reilly, 1996), increasing market share, decreasing costs, raising flexibility and the accelerating new product development, which are similar to those reported in previous research, which, in general terms, found positive effects on firm performance (Prange and Verdier, 2011; Rialti et al., 2018; Peng et al., 2019a, b). However, due to some tension between the two capabilities, there must be some trade-offs between them (Drnevich and Kriauciunas, 2011). This study inferred that the relationship among exploration, exploitation and firm performance system is a curvilinear (inverted U-shaped) relationship. Our findings also showed that exploration have a curvilinear (inverted U-shaped) relationship with firm performance, and that exploitation have a U-shaped relationship with firm performance. As stated by Slotegraaf et al. (2003); He and Wong (2004), and Stock and Reiferscheid (2014), both exploration and exploitation are important dilemmas for the survival and development of SMEs. Therefore, SMEs should have both exploration and exploitation in order to reduce the possibility of falling into the capability trap or failure trap. With regard to long-term development, new capabilities are needed if the firm is going to survive. The development of new products, production technologies and marketing modes will bring new value to customers, which can help manufacturers to expand in existing or new markets, and so exploration are vital. Exploitation has a U-shaped relationship with firm performance, although this hypothesis is inconsistent with the results of this study. This can be interpreted by the “threshold effect,” and thus that relationship between exploitation and firm performance has two stages. First, in the early stages of development and marketing, exploitation is helpful for manufacturers to achieve higher profits and performance. But in the mature stage of the product life cycle, the firm’s exploitative resources will be significantly consumed, which may lead to a financial burden and have negative effects on performance. In fact, the use of multi-exploitative strategies in a firm’s life cycle often leads to extra control and coordination costs. After entering the second stage, firms will invest more resources in exploitation, and then pass the threshold between application activity and performance, thus achieving an economy of scale (Lee and Rugman, 2012), as seen in a differentiated production process, simplified sales process and reduced purchasing cost, which can all help firms to achieve better performance.

In terms of ambidexterity, this study referred to the work of He and Wong (2004); Cao et al. (2009), and Rialti et al. (2019), and verified the relationship between ambidexterity and firm performance. The results showed that ambidexterity and firm performance have a significantly positive relationship, which means that exploration and exploitation may sometimes have a competitive relationship with regard to organizational resource, or instead a complementary relationship (O’Reilly and Tushman, 2013; Junni et al., 2013). Moreover, the measurement of ambidexterity in this study is consistent with previous studies (Gibson and Birkenshaw, 2004; He and Wong, 2004; Peng et al., 2019a, b), it adopted the approach of combined dimension, but it is differentiated with regard to analysis comparing with the previous hierarchical regression model, it adopted PLS-SEM to verify the role of ambidexterity in the model, which contributed to the research approach. Firms with high exploration can improve their efficiency with regard to exploring new knowledge and developing new products, as well as expanding markets (Rialti et al., 2018). This is because by repeatedly using existing knowledge and resources, managers can have a clearer understanding of their firms and their situations (Kristal et al., 2010). In this case, the firms may be more powerful in controlling the construction of existing knowledge and resources, and the successful development of new products and technologies. The research results also verify that the moderating effect of market orientation has positively correlated to relationship between ambidexterity and firm performance (Menguc and Auh, 2008; Peng et al., 2019a). The establishment of a market-oriented culture could provide firms with a direction to both better perceive and interpret their exploration and exploitation, and thus to facilitate a complementary relationship between them.

### Practical Implications

This discussion draws forth the managerial implications of this study, mainly concerning how SMEs improve their dynamic capability and firm performance through organizational learning process. First, our results found that internal and external social capital positively strength exploration and exploitation. This implies that acquiring knowledge from external firms and integrating knowledge from internal members are effective ways to improve exploration and exploitation. Besides, as knowledge has the characteristics of being accumulative, specific, complex and tacit, these features will have different influences on a firm’s competitors, and have transfer boundaries and limitations to some degree. Therefore, this study suggests that managers can shorten development cycles and lower exploration costs through acquiring knowledge from partners and improving their exploration. In an industrial network, the related firms might ignore customers’ customers or suppliers’ suppliers. SMEs with well-structured embeddedness may occupy a superior position and receive more explicit knowledge from customer or supplier groups (Zaheer and Bell, 2005; Lin et al., 2016).

Moreover, internal social capital plays a significant role in improvement of dynamic. Our findings suggest managers to encourage to accumulate development requirements of exploration and exploitation through formal (e.g., integrated information system, electronic communication system, internet and business intelligent) and informal (inter-departmental personal relationships, banquets and information-sharing social network) knowledge-processing mechanisms, thus improving knowledge base of dynamic capability. In other words, managers can construct an information exchange platform within the organization to cultivate members’ abilities with regard to the interpretation and demonstration of external information and knowledge, thus lowering internal cognitive differences. With the organizational learning process, the accumulation of new knowledge is helpful for updating existing knowledge stores and changing the current knowledge structure, so that enterprises can constantly enhance both new and existing capabilities to facilitate organizational growth.

Third, SMEs with ambidexterity of exploitation and exploration have better firm performance. Therefore, this study suggests that SMEs develop new products, technologies and knowledge use efficiency. For instance, numerous SMEs strive for internalization in pursuit of market expansion, and to break through time and space constraints and build network cooperating teams with customer relations and supply chain relations through IT-based networking such as internet, intelligent office, cloud computing technologies, so as to achieve development synergy of exploitation and exploration that is able to promote their productivity and value creation at the lowest cost (Rialti et al., 2018, 2019).

Fourth, the results of this study showed that market orientation will enhance the positive effects of ambidexterity on firm performance. Market-driven SMEs with a market-oriented culture will transfer, absorb and reserve knowledge through an inside-out, cross boundary process, and engage in new product and technology development (Anwar, 2008). Furthermore, SMEs using an outside-in flow capacity are committed to cost control, financial management and manufacturing processes, which are consistent with exploitation. From this we can know that firms with a high market orientation will facilitate integration among exploration and exploitation, and provide the basis for the development of exploration with fixed profits to achieve complementary effects.

### Research Limitations and Suggestions for Future Studies

As a result, this study put forward the limitations of the study and the direction of future study to make the DC theory can be built more integrated. This study has three limitations that should be prompted in further research. First, due to the cross-sectional nature, this study cannot test how firms cascade their exploration into exploitation. To conduct such issues, further studies could employ longitudinal data to verify the impact of evolution of social capital on firm performance.

Second, although this study well-established constructs to develop research framework based on previous literatures, such exploration/exploitation and inter-/intra-firm social capital, are still valid and expected to influence firm performance as well. Thus, including innovation-specific and environmental determinants of DCs and organizational ambidexterity and exploring correlation among them will be an interesting further research.

Third, Huge cultural diversities play a vital role in the SMEs. But SMEs in only one country are involved this study, and the impact of the cultural diversity is not considered. Therefore, future researchers are suggested to include SMEs of different countries in their studies to ensure the universality of the research results.

## Data Availability Statement

The raw data supporting the conclusions of this article will be made available by the authors, without undue reservation.

## Ethics Statement

The studies involving human participants were reviewed and approved by Ethics Committee in University of Taipei. The patients/participants provided their written informed consent to participate in this study.

## Author Contributions

LZ, LS, and MP contributed to the ideas of educational research, collection of data, and empirical analysis. MP, LS, H-YY, and K-HL contributed to the data analysis, design of research methods, and tables. MP and MA participated in developing a research design, writing, and interpreting the analysis. All the authors contributed to the literature review and conclusions.

## Conflict of Interest

The authors declare that the research was conducted in the absence of any commercial or financial relationships that could be construed as a potential conflict of interest.

## Appendix

**APPENDIX 1:** Scales.

Construct	Variables	Items
Dynamic Capability	Exploration	Introduce new generation of products.
		Extend product range.
		Open up new markets.
		Enter new technology fields.
		Innovations in marketing techniques.
	Exploitation	Improve existing product quality.
		Improve production flexibility.
		Reduce production cost.
		Improve yield or reduce material consumption.
Firm Performance	Organizational effectiveness	Product quality.
		New product success rate.
		Customer retention rate.
	Growth/share	Sales.
		Growth rate.
		Targeted market share.
	Profitability	Return on Equity (ROE).
		Gross margin.
		Return on Investment (ROI).
Market Orientation	Customer orientation	Meet patients to find what they will need in the future.
		Interact with patients to learn how to serve them better.
		Fast in detecting changes in patients’ preferences.
		Pay attention to patient complaints.
		Immediate corrective action to services customers are unhappy with.
		Front Office personnel discuss patients’ future needs with other departments.
		Regular dissemination of data about patient satisfaction/dissatisfaction at all levels in the organization.
	Competitor orientation	Departments independently generate market intelligence on competitors.
		Fast at detecting major shifts and trends in the industry.
		Fast in alerting other departments of important findings about competitors.
		Respond immediately to promotional campaigns by Competitors.
		Quick response to changes in competitors’ pricing Structures.
	Environmental scanning	Informally collect industry information.
		Periodically review the likely effect of changes in the business environment.
		Quarterly interdepartmental meetings to discuss market trends and developments.
		Whole business unit is aware of significant events occurring in the major market.
		Minimal communication between marketing and service departments concerning market developments.
	Strategy implementation	Share survey results with influencers of our end users’ Purchase.
		Good coordination among departments in the organization.
		Able to implement marketing plan on time.
		Concerted effort by all departments involved in modifying products/services customers would like to modify.
	New services development	In-house research.
		Survey end users to assess quality of product and service Offerings.
		New product development driven by the principles of patients’ needs.
		Periodically circulate information on patients.
Inter-firm Social Capital	Bridging ties	Managerial resource.
		Emotional support.
		Partners’ management time.
	Joint problem solving	We work with our main partners to help solve each other’s problems.
		Our main partners work with us to overcome difficulties.
		We are jointly responsible with our main partners for getting things done.
	Shared value	Partners’ systems in firms tailored.
		Partners monitor established procedures.
		Our systems meet partners requirement.
		We incorporate partners philosophy.
Intra-firm Social Capital	Information-based mechanism	We have integrated software applications.
		We have integrated information systems.
		We have electronic communications systems.
		We have interconnected computer systems.
		We have databases to share information.
	People-based mechanism	A committee that meets regularly to plan and integrate activities.
		Meetings of managers from different locations.
		Liaison personnel to integrate activities.
		Personal contacts among managers from different locations.
		Transfers of people.
	Trust	Members know each other.
		Members never feel cheated.
		Members do not expect to harm others’ interests.
		The most powerful members do not pursue their own interests for the cost.
		Members know each other’s weaknesses but do not exploit them to their.
	Shared vision	Our unit shares the same ambitions and vision with other units at work.
		People in our unit are enthusiastic about pursuing the collective goals and missions of the whole organization
